# Serotonin 2B Receptor Antagonism Prevents Heritable Pulmonary Arterial Hypertension

**DOI:** 10.1371/journal.pone.0148657

**Published:** 2016-02-10

**Authors:** James D. West, Erica J. Carrier, Nathaniel C. Bloodworth, Alison K. Schroer, Peter Chen, Larisa M. Ryzhova, Santhi Gladson, Sheila Shay, Joshua D. Hutcheson, W. David Merryman

**Affiliations:** 1 Division of Allergy, Pulmonary, and Critical Care Medicine, Vanderbilt University Medical Center, Nashville, Tennessee, 37232, United States of America; 2 Department of Biomedical Engineering, Vanderbilt University, Nashville, Tennessee, 37232, United States of America; Nippon Medical School Graduate School of Medicine, JAPAN

## Abstract

Serotonergic anorexigens are the primary pharmacologic risk factor associated with pulmonary arterial hypertension (PAH), and the resulting PAH is clinically indistinguishable from the heritable form of disease, associated with BMPR2 mutations. Both BMPR2 mutation and agonists to the serotonin receptor HTR2B have been shown to cause activation of SRC tyrosine kinase; conversely, antagonists to HTR2B inhibit SRC trafficking and downstream function. To test the hypothesis that a HTR2B antagonist can prevent BMRP2 mutation induced PAH by restricting aberrant SRC trafficking and downstream activity, we exposed BMPR2 mutant mice, which spontaneously develop PAH, to a HTR2B antagonist, SB204741, to block the SRC activation caused by BMPR2 mutation. SB204741 prevented the development of PAH in BMPR2 mutant mice, reduced recruitment of inflammatory cells to their lungs, and reduced muscularization of their blood vessels. By atomic force microscopy, we determined that BMPR2 mutant mice normally had a doubling of vessel stiffness, which was substantially normalized by HTR2B inhibition. SB204741 reduced SRC phosphorylation and downstream activity in BMPR2 mutant mice. Gene expression arrays indicate that the primary changes were in cytoskeletal and muscle contractility genes. These results were confirmed by gel contraction assays showing that HTR2B inhibition nearly normalizes the 400% increase in gel contraction normally seen in BMPR2 mutant smooth muscle cells. Heritable PAH results from increased SRC activation, cellular contraction, and vascular resistance, but antagonism of HTR2B prevents SRC phosphorylation, downstream activity, and PAH in BMPR2 mutant mice.

## Introduction

Pulmonary arterial hypertension (PAH) is a disease in which a gradual increase in pulmonary vascular resistance eventually leads to right heart failure and death. There are no clinically available disease-modifying therapies for PAH. The strongest epidemiologic risk factor is use of serotonergic anorexigens [1]. There have been two epidemics of serotonergic anorexigen-induced PAH; aminorex in the 1970s [2] and dexfenfluramine in the 1990s [3,4].

Several mouse models have been developed to examine the role of serotonin signaling in the onset of PAH. Mice with knockout for serotonin transporter (5HTT) [5,6], 1B [7] or 2B [8] receptors (HTR1B and HTR2B) are protected against hypoxic pulmonary hypertension. While excellent work has been done demonstrating that increased serotonin signaling is responsible for the onset of PAH in patients taking anorexigenic drugs, essentially no work has been done previously to mechanistically link signaling at the level of the receptor to physiologic outcomes. A recent comprehensive review left the space between the cell surface and the nucleus essentially blank [9].

The strongest heritable risk factor for development of PAH, independent of serotonergic drugs, is presence of a mutation in the type 2 receptor for the BMP pathway (BMPR2), present in the large majority of familial cases. Mice expressing human-derived BMPR2 mutations develop PAH within a few weeks [10,11]. In both mice and humans with BMPR2 mutation, penetrance is incomplete, with lifetime risk of overt disease of about 20–25% in patient families [12], and 30–50% in BMPR2 mutant mice after 6 weeks of transgene activation [10]. Although serotonin has been shown to increase penetrance in BMPR2-deficient mice [13], the mechanism has never been explored. Anorexigen-associated PAH is clinically indistinguishable from idiopathic or heritable PAH, suggesting that common mechanisms downstream of the cell-surface receptors mediate all forms of the disease.

The mechanism underlying PAH of any kind is unknown; however, heritable and drug-induced PAH share some common features. Both HTR2B and BMPR2 receptors interact directly with the tyrosine kinase, SRC. SRC binds to the cytoplasmic tail of BMPR2 [14], and BMPR2 mutation leads to increased SRC phosphorylation and downstream activity [10,15]. Likewise, agonism of HTR2B, by either serotonin or metabolites from anorexigens, does the same [16,17]. Therefore, HTR2B and BMPR2 likely have no effect on one another, but their functionality significantly and independently alters SRC activity, which appears to be a key component in the development of PAH. Further, we previously found that antagonism of HTR2B in heart valve cells inhibits SRC translocation after its phosphorylation [18]; this is important since valvular disease often accompanies drug-induced PAH [19]. Taken together, we hypothesized that antagonism of HTR2B may be able to prevent heritable PAH through the regulation of SRC by preventing its downstream activities, but not its phosphorylation. To test this hypothesis, we examined the ability of a specific small molecule HTR2B antagonist, SB204741 [18,20,21], to prevent PAH in mice with BMPR2 mutation.

## Materials and Methods

### BMPR2 Mutant Mice

Rosa26-Bmpr2^R899X^ mice express the patient-derived R899X mutation in BMPR2 in all tissues when induced with doxycycline. When BMPR2^R899X^ transgene is induced in adult mice for six weeks of activation, approximately 50% will develop PAH as defined by right ventricular systolic pressures (RVSP) above the normal range [10]. Adult male (10–14 weeks of age at start) BMPR2 mutant mice (38 Rosa26-rtTA2M2 X TetO_7_-Bmpr2^R899X^ mice and 16 Rosa26-rtTA2M2 only controls) on an FVB/N strain background were fed doxycycline at 0.2g/kg in western diet (Bioserv) for 6 weeks. Mice were kept at a maximum of 5 mice per cage on corn cob bedding and monitored twice weekly for injury or illness (lack of grooming, hunched posture, etc.) Pulmonary hypertension in these mice does not proceed to the point where the mice show signs of illness at the time point chosen. After two weeks, osmotic pumps (Alzet 1004) containing either SB204741 in 50% DMSO/50% water or vehicle with the same DMSO/water formulation were implanted, and delivered SB204741 at 1 mg/kg/day or vehicle for the final four weeks. A similar dose (i.e. 3 mg/kg/day) has been used previously to successfully attenuate liver fibrosis in mice [20]. Mice were then placed under surgical anesthesia (Avertin) and RVSP measured through a catheter introduced into the right heart through the right jugular vein in a closed-chested procedure, as previously described [22]. After sacrifice by exsanguination under tribromoethanol or pentobarbital anesthesia, tissues were collected for further analysis. All procedures were approved by the Vanderbilt institutional animal care and use committee (IACUC).

### Histology & Western Blots

Lungs were flushed with 5 ml PBS introduced through the right ventricle and allowed to flow out through a cut in the left atria to remove blood, then inflated with 0.8% low melt agarose and formalin fixed. Staining for CD45R was with BD Pharmingen # 550286 at 1:100. An observer blinded as to groups counted numbers of CD45 positive cells per field in 10 random 20x fields in each of four mice per group.

Downstream SRC targets, p130Cas (CAS) and caveolin-1 (CAV1) were primarily quantified as a measure of SRC activity. Antibodies used for Western blots were: SRC and pSRC (Cell Signaling, #s 2110 and 2101, 1:1000), CAS and pCAS (Abcam, # ab89459 and Cell Signaling, # 4015, 1:1000), CAV1 and pCAV1 (BD Transduction Laboratories, #s 610684 and 611338, 1:1000 and 1:2000), Smad1 and pSmad1 (Cell Signaling, #s 6944 and 9511, 1:1000). All phosphorylation proteins were normalized to their respective total protein and β-actin (i.e. pSRC/SRC/β-actin).

### Gene Expression Analysis

Mouse Genome 430 2.0 microarrays (Affymetrix, Foster City, CA) were performed on homogenized whole lung tissue, as previously described [23]. Each array consisted of a pool of 3 mice, and two arrays were used per condition. Array results were submitted to the NCBI gene expression and hybridization array data repository (GEO, http://www.ncbi.nlm.nih.gov/geo/) accession number (pending).

Preprocessing of all Affymetrix cel files was carried out using the RMA algorithm. Hierarchical clustering of both samples and genes, and principal components analysis, was performed using algorithms within JMP Pro 11.0 (SAS Institute). Statistical analysis of overrepresented gene ontology groups was performed using Webgestalt [24].

### Measurements of pulmonary arteriole wall elastic modulus

Atomic force microscopy (AFM) of whole tissue sections was adapted from previously published techniques for mouse heart valve leaflets [25] and lungs [26]. Lungs from mice with or without a doxycycline-inducible mutation in the BMPR2 receptor were isolated, embedded with Optimal Cutting Temperature compound, and sectioned after the mice were treated for 4 weeks with SB204741 or DMSO vehicle and hemodynamically phenotyped as described. Lung sections were stained with FITC conjugated rat anti-mouse CD31 (BD Biosciences), Cy3 conjugated mouse monoclonal α smooth muscle actin (αSMA, Sigma), and DAPI. Sections were immersed in PBS and CD31 and αSMA positive vessels less than 100 μm in diameter were identified with a Nikon Eclipse Ti microscope. Identified vessels were then scanned using a Bioscope Catalyst AFM at a scanning frequency of 0.25 Hz and a scan window size of 7–10 μm. A total of 5–7 vessels were scanned per animal from two sections of lung, with each vessel scanned in two separate regions.

The data presented are representative of single scans, consisting of 16384 individual measurements (128x128) spanning an approximately 10–20 um^2^ area along the vessel wall. The median value for each scan (in kPa) is used as a representative measurement for the entire scan window. This analysis method for AFM data for both tissues and biomaterials has been previously validated [27,28] and the results scale well with bulk modulus measurements [25].

### BMPR2 mutant Cells

Cells used were derived from Immortomouse X Rosa26-rtTA2 X TetO_7_-BMPR2^R899X^ or Immortomouse X Rosa26-rtTA2 X TetO_7_-BMPR2^delx4+^ triple transgenic mice. The immortomouse contains a transgenic insertion of the SV40 large T antigen, tsA58, under control of an interferon-inducible promoter [29]. When cells are grown at 33°C and interferon is added, the transgene is activated and the cells are immortalized and proliferate freely; at 37°C, this transgene is inert. The immortomouse therefore produces cells which proliferate as though they were immortalized at 33°C, but revert to a more normal phenotype when cultured at 37°C. Immorto-BMPR2 mutant pulmonary endothelial and smooth muscle cells were collected from adult mice as previously described [30].

### SRC and tubulin motility analysis

Immortalized microvascular endothelial and smooth muscle cells, with or without mutant BMPR2 induced with 300 ng/mL doxycycline, were co-transfected with fluorescently labeled SRC and Tubulin and treated with 1 μM SB204741 or DMSO vehicle. SRC and Tubulin motion was visualized with a Nikon Eclipse Ti confocal microscope for 15 min in four separate focal planes. Videos were analyzed in MATLAB using a custom Eulerian motion analysis algorithm to determine total motion by assessing differential changes in pixel intensity for each cell [18]. SRC motion was weighted to the perinuclear region of each cell to adjust for anomalies induced by changes in cell edge positioning, and total motion was normalized to total pixel intensity and averaged across the four focal planes visualized for each cell.

### Total and active TGF-β1 assay

Total and active TGF-β1 was assayed as described previously [31]. Briefly, immortalized microvascular endothelial and smooth muscle cells with or without a doxycycline inducible BMPR2 receptor mutation were plated in 6 well plates at 40,000/cm^2^ and cultured for 24 hours with 300 ng/mL doxycycline to induce expression of the transgene. After 24 hours, media was collected and prepared as follows. For measurements of activated TGF-β1, a 1:1 dilution of media to serum-free Dulbecco’s modification of Eagle’s medium (DMEM, Corning CellGro) was prepared, and for measurements of total TGF-β1 media was heated to 100°C for 3 minutes (to activate latent TGF-β1) and diluted 1:10 with serum-free DMEM. The prepared media was added to cultures of transformed mink lung cells (TMLCs) transfected with a luciferase reporter gene with a TGF-β1 specific promoter and incubated for 18 hours. After incubation, cells were lysed and the lysate transferred to a white 96 well plate. Luminescence intensity was measured using a BioTek Synergy HT plate reader after automatically dispensing luciferase substrate from the Promega Luciferase Reporter kit. Luminescence intensity was correlated with TGF-β1 concentration with the aid of a standard curve.

### Collagen gel contractility assay

The collagen gel contractility assay was adapted from previously published work [32]. A collagen gel solution consisting of 8:1:1 parts bovine collagen (Advanced BioMatrix PureCol), 10x Dulbecco’s phosphate buffered saline (DPBS, Gibco), and 0.1 M NaOH was prepared and the pH adjusted to 7.4. 200 μL of gel solution was dispensed to 1.27 cm diameter Teflon rings (Seastrom Manufacturing Company) and the gel allowed to crosslink for 1.5 hours at 37°C. The top of the gel was seeded with a 200 μL of a cell suspension containing 40,000 immortalized microvascular endothelial and smooth muscle cells with or without a doxycycline inducible BMPR2 receptor mutation and allowed to settle for 30 minutes. The Teflon rings were removed and media added containing 300 ng/mL of doxycycline and the cells treated with either 1 ng/mL TGF-β1 (porcine, R&D Systems Inc.), 1 μM SB204741 (Tocris), both, or neither. Gels were imaged using a dissection microscope (Olympus) at 30 minutes and 72 hours after seeding, and the treatment media was changed every 24 hours. Gel size was determined using ImageJ (National Institutes of Health).

### Statistical methods

Statistics were performed using multiple factor ANOVA (+/- BMPR2 mutation, +/- SB204741), with Fisher’s exact test or Holm-Sidak post hoc test for comparisons between individual groups. Statistics were performed within JMP Pro 11.0 (SAS Institute).

## Results

### HTR2B Antagonism Prevents PAH in BMPR2 Mutant Mice

**Fig 1:**
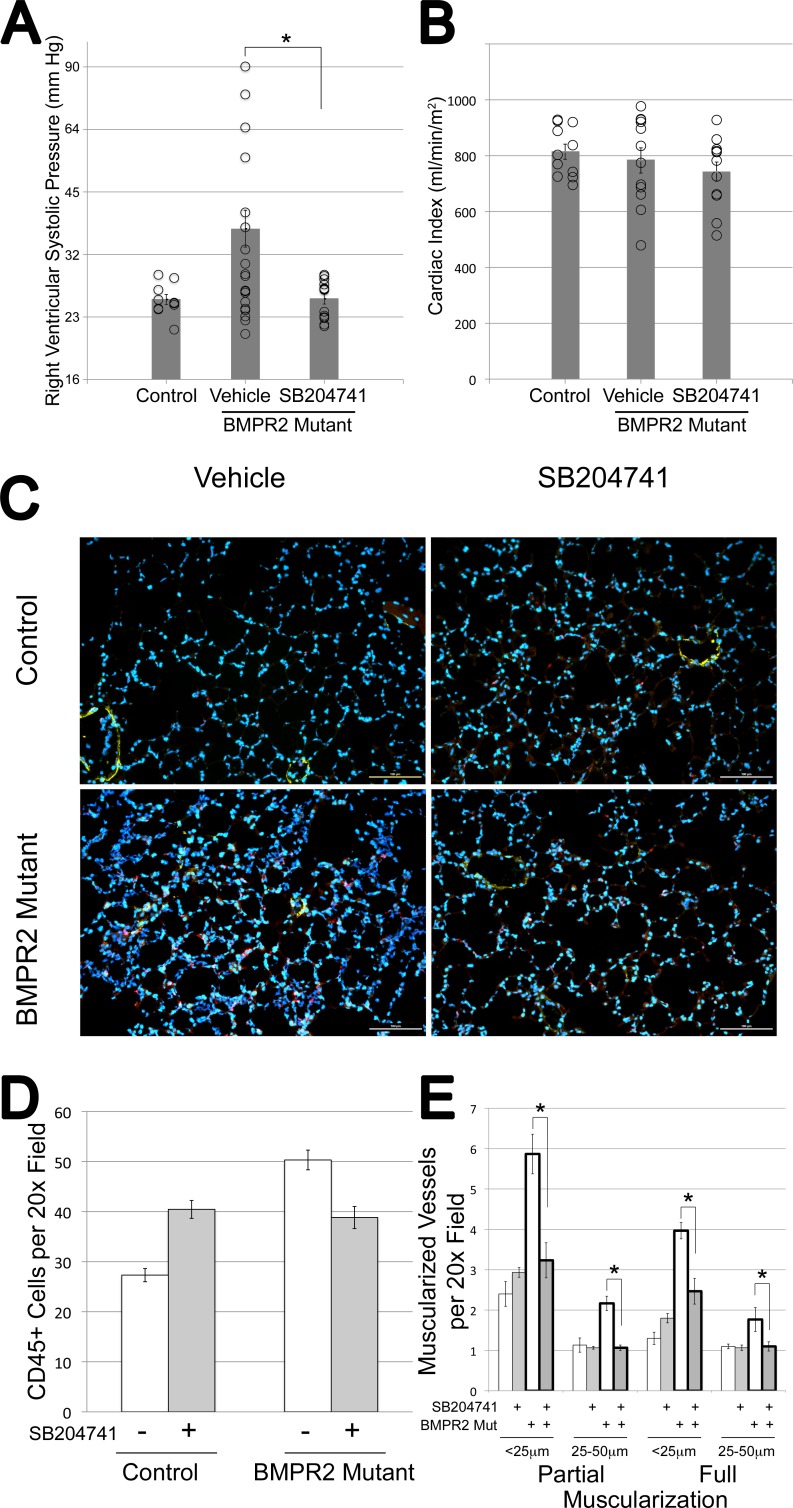
**(A)** Right ventricular systolic pressures are significantly elevated in BMPR2 mutant mice with six weeks of transgene activation using doxycycline at 1g/kg in western diet; this elevation was prevented through administration of SB2014741 in pumps for the final four weeks. Circles represent individual mice; columns are averages of log_2_-transformed values; error bars are SEM. SB204741 did not affect control mice; both vehicle and treated mice are included in the control column as left and right groups of circles, respectively. **(B)** Cardiac Index does not change between groups, measured as cardiac output in ml/minute as determined by echocardiography divided by body surface area in square meters. **(C)** Immunoflourescence staining for CD45 in a 10x field of distal alveoli in agarose-inflated lungs. An increase in CD45+ cells is immediately apparent with BMPR2 mutation and with SB204741 treatment in controls. **(D)** BMPR2 mutant mice have ~2x the inflammatory cells per field at baseline, but SB204741 treatment has divergent effects on inflammatory cells in control and BMPR2 mutant mice, causing significant increases and decreases respectively (* = p<0.05, § = p<0.01). **(E)** BMPR2 mutant mice have roughly twice the numbers of partially and fully muscularized vessels per field for small and medium sized vessels; this is substantially normalized by SB204741 (* = p<0.01).

Wild-type and BMPR2 mutant mice were treated with the HTR2B antagonist SB204741 for the last four weeks of a six week transgene activation. While vehicle-treated mice developed elevated RVSP at about 50% penetrance, mice treated with SB204741 have pressures indistinguishable from controls (**Fig 1A**). This rescue of RVSP was not due to suppressed cardiac output, as cardiac index was maintained (**Fig 1B**). Note that BMPR2 mutation in both mice and humans leads to right ventricular dilation under pressure, rather than hypertrophy [33], and so Fulton index was not assessed.

SB204741 did not impact either weight gain or blood glucose in these mice (**S1A and S1B Fig**). Lung sections from BMPR2 mutant mice had increased infiltrating cells, as previously reported [34], with the infiltrating cells being made up in large part of CD45+ inflammatory cells. This increase in infiltrating cells was reduced by SB204741 treatment in BMPR2 mutant mice, but increased by treatment in control mice (**Fig 1C, 1D and S1C Fig**), a pattern which will reoccur with many of the following results. HTR2B antagonism also reduced both partial and full muscularization of small pulmonary arteries in BMPR2 mutant mice, without affecting muscularization of vessels in control animals (**Fig 1E**). Partial muscularization is defined as actin staining surrounding less than 75% of the vessel perimeter, and is usually indicative of muscle spiraling along a vessel rather than completely surrounding it (full muscularization).

### HTR2B Antagonism Reduces Vascular Stiffness in BMPR2 Mutant Mice

**Fig 2:**
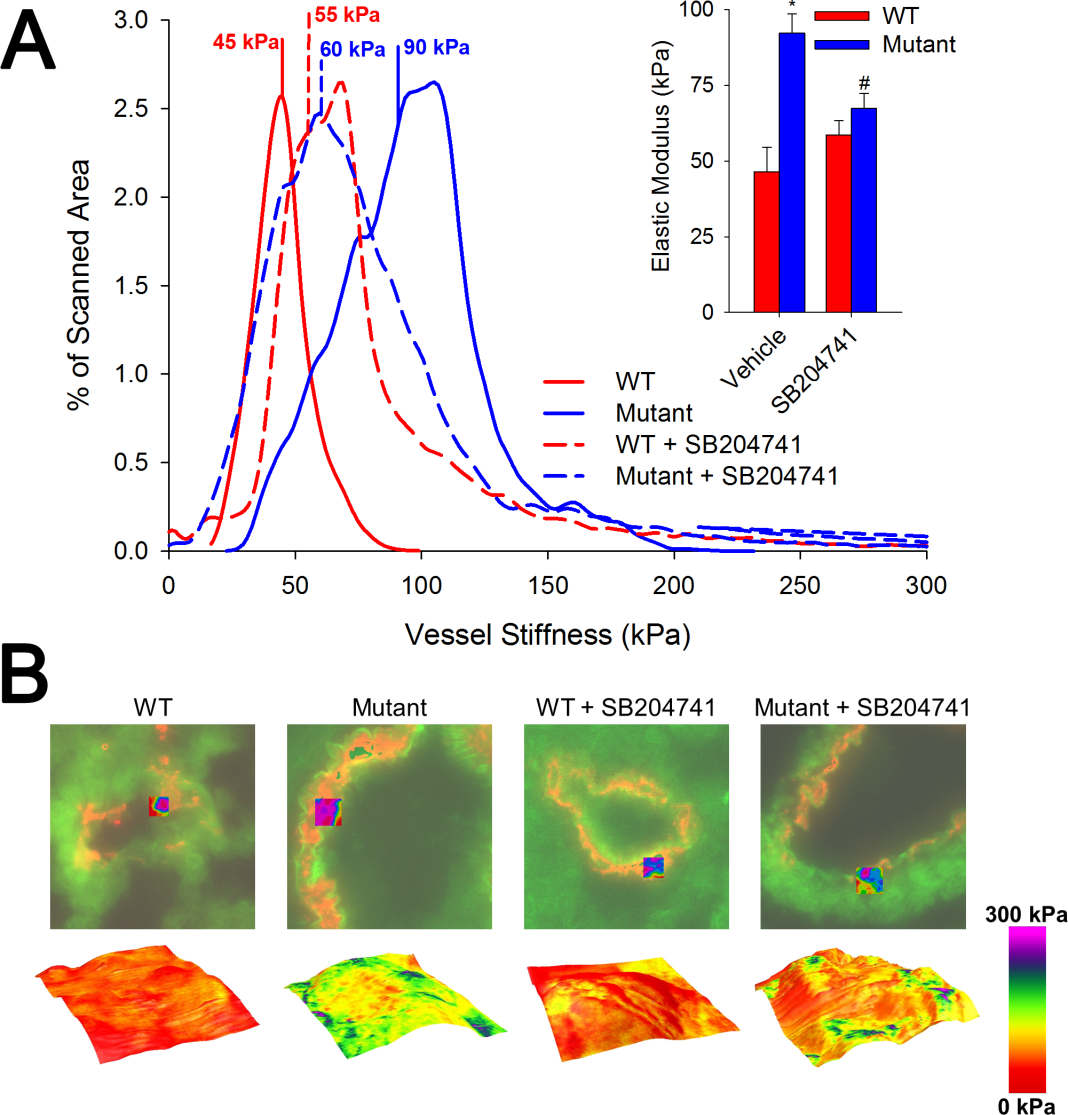
SB204741 prevents arteriole wall stiffening in BMRP2 mutant animals. **(A)** In mutant animals treated for 4 weeks with SB204741, the average elastic modulus is significantly lower than their untreated counterparts. The histogram shows representative vessels with a Gaussian fit of elastic modulus distributions and calculated median values. Inset bar graph shows median stiffness values. **(B)** Fluorescent images (green = CD31; red = αSMA), with a topographical map of vessel height overlaid with a colorimetric representation of the elastic modulus. Values in the graph in (A) are expressed as mean ± standard error. n = 3 per group, *p<0.05 compared to WT, #p<0.05 compared to vehicle treated.

Although BMPR2 mutant mice have occlusion of small arteries as determined by microCT [10], particularly at branch points, increase in RVSP in these mice may be driven by increased vascular stiffness. Here, we used atomic force microscopy (AFM) to assess lung sections, and found that small vessels in BMPR2 mutant mice have twice the stiffness of control animals, with a median elastic modulus of 90 kPa as compared to 45 kPa. This stiffness is significantly normalized when mice are treated with HTR2B antagonist (**Fig 2**). The stiffness distribution presented may be bimodal, possibly indicative a heterogeneous deposition of ECM components in the vessel wall. Increased vascular stiffness has been hypothesized to be the pathologic feature of human PAH central to etiology [35,36], and so this prevention has high prognostic significance for translation potential.

### HTR2B Antagonism Reduces SRC Activity and Motion in BMPR2 Mutant Mice and Cells

**Fig 3:**
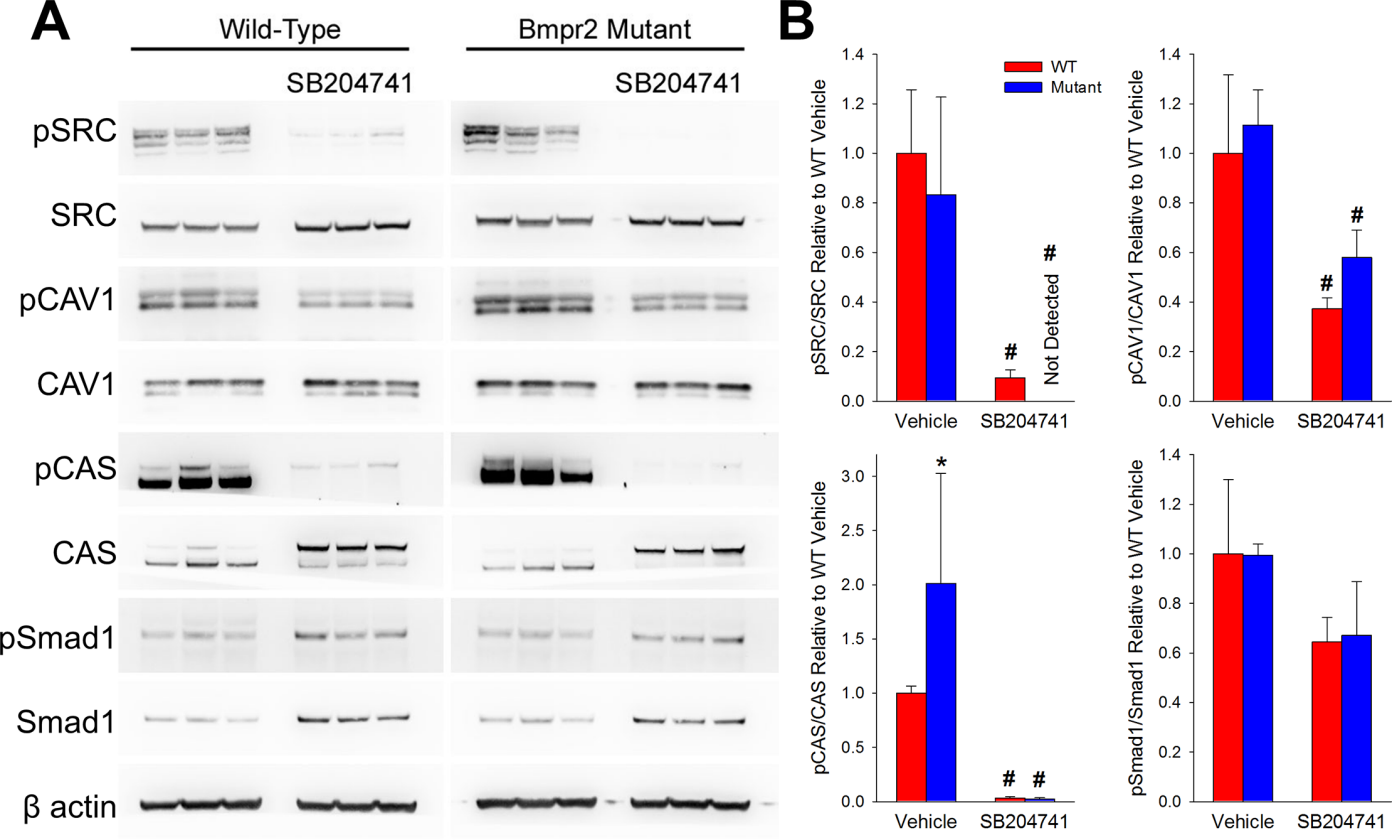
SB204741 reduces SRC phosphorylation and downstream activation through reduction of motility of SRC. **(A)** Western blots from BMPR2 mutant or WT mice whole lung treated with SB204741 or vehicle. BMPR2 mutants show increased phosphorylation of SRC target CAS; SRC activity and phosphorylation is reduced with SB204741 treatment. **(B)** Densitometry for pSRC, pCAS, and pCAV1 phosphorylation. Values are normalized to total protein and β-actin (i.e. pSRC/SRC/β-actin). n = 3, *p<0.05 compared to WT, #p<0.05 compared to vehicle treated.

Antagonism of the HTR2B receptor has been shown to reduce SRC’s downstream activity by restricting its intracellular trafficking without reducing phosphorylation [18] and BMPR2 mutation has been previously found to increase SRC activity [15,37]. Therefore, we sought to determine if SB204741 could reduce SRC downstream activity in BMPR2 mutant mice. By western blot on lungs from mice, we found phosphorylation of SRC and its downstream target CAS were increased in BMPR2 mutants, with SRC, CAS, and CAV1 phosphorylation significantly inhibited with chronic SB204741 treatment. Smad1 phosphorylation was not altered due to SB204741 (**Fig 3A and 3B**).

**Fig 4:**
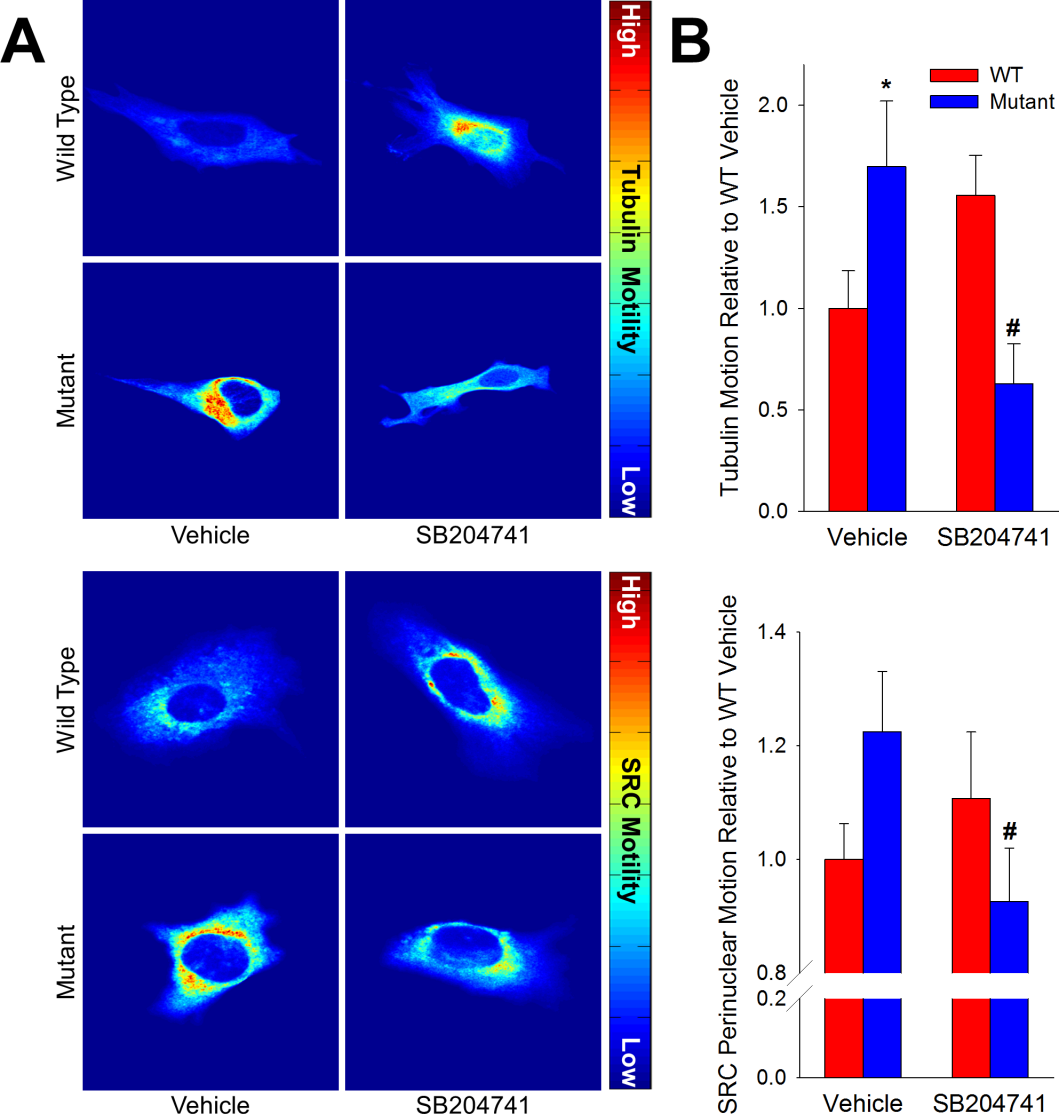
SB204741 restricts motion of pSRC. **(A)** SB204741 reduces tubulin and perinuclear SRC motility, both of which are increased in mutant microvascular endothelial cells. Eularian analysis of fluorescently labeled tubulin and SRC in endothelial cells shows elevated motility in vehicle treated mutant cells, as well as a significant decrease in motility in mutants cells treated with SB204741. Values are expressed as mean ± standard error. n = 5–10, *p<0.05 compared to WT, #p<0.05 compared to vehicle treated.

To determine whether HTR2B inhibition affected SRC translocation, we motion-tracked fluorescently labeled tubulin and SRC in transfected live pulmonary microvascular endothelial cells, cultured from wild-type or BMPR2 mutant mice, and converted the motion to a heat map. We found that at baseline, BMPR2 mutant cells had higher tubulin and SRC motion than did wild-type cells, but these were normalized with SB204741 treatment. Conversely, wild-type cells had these motions increased (but not significantly) with SB204741 treatment (**Fig 4A and 4B**). Once again, this contrast between drug effect in BMPR2 mutant and wild-type cells suggests that the drug is impacting a pathway fundamentally altered by BMPR2 mutation.

### HTR2B Modulates Muscle Contractility Genes in BMPR2 Mutant Mice

**Fig 5:**
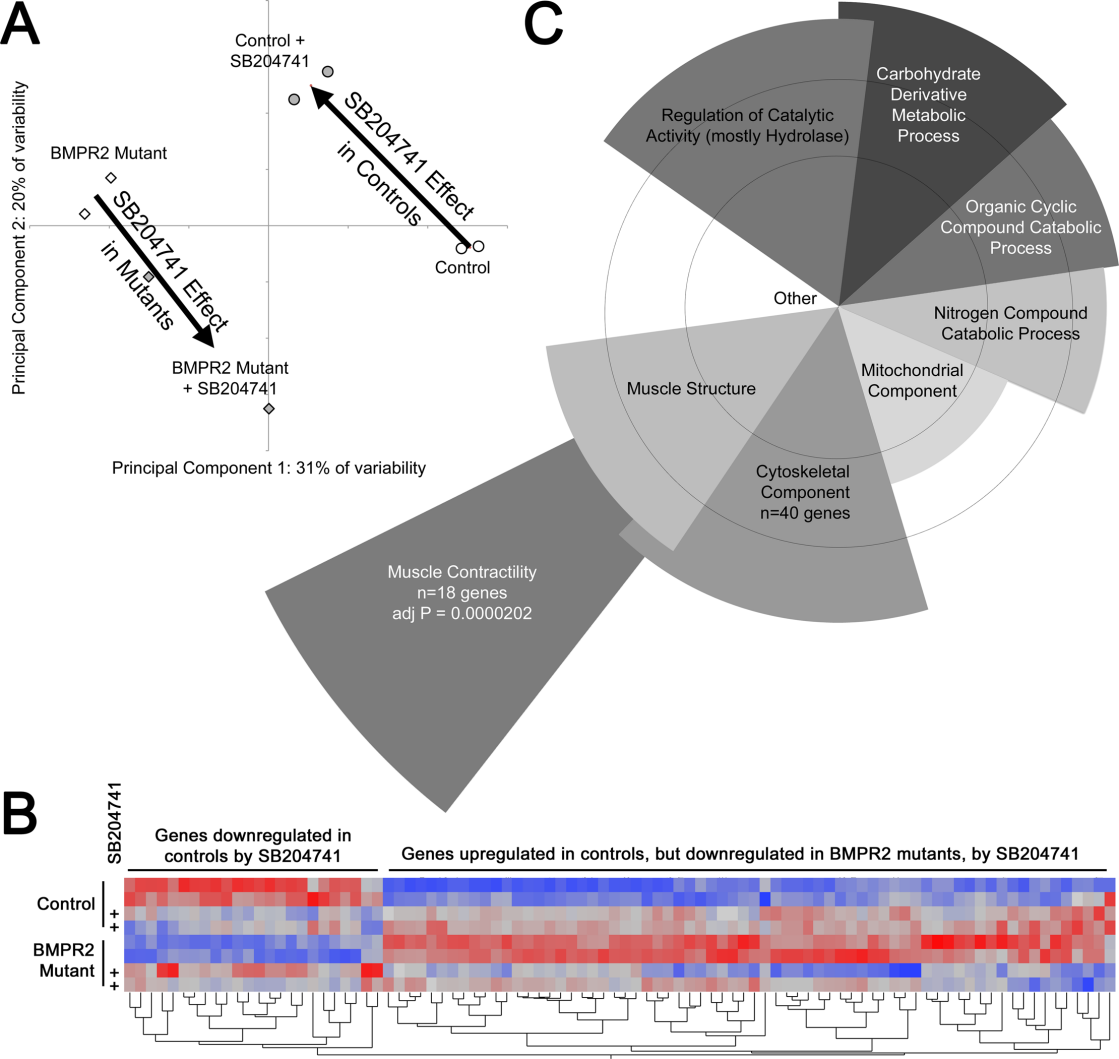
**(A):** Principal components analysis found a strong difference between BMPR2 mutants and controls along Principal Component 1 (PC1). Treatment with SB204741 caused nearly opposite changes in PC vector in control and mutant mouse lungs (large arrows). Circles and diamonds refer to individual arrays for control and BMPR2 mutants respectively: open and filled shading are for vehicle and SB204741 treatment respectively. **(B):** Heat map of normalized gene expression for 100 genes most affected by SB204741 treatment. Each column is a gene, with rows treatment/genotype groups. Red corresponds to high expression and blue to low. In general, SB204741 eliminates differences between control and BMPR2 mutant mice, by moving gene expression in opposite directions (BMPR2 mutants become more like controls, but controls become more like BMPR2 mutants). **(C):** Representative examples of significantly overrepresented gene ontology groups. Angular width of each wedge is proportional to the number of genes altered by SB204741 in the group as a fraction of the 234 with a 95% confidence of change of over 20%. Radius is proportional to–log of the p-value (so longer is more significant). Circles correspond to multiple comparisons adjusted p = 0.05 and p = 0.01. Overlap is approximate, and demonstrates that most genes belong to more than one ontology group (lower level ontology groups not shown).

To further examine molecular changes in BMPR2 mutant mice caused by chronic HTR2B antagonism, Affymetrix gene expression profiling was performed on pools of lung RNA from mice with and without BMPR2 mutation and with and without SB204741 treatment. Principal components analysis found that all four groups were well separated, but with changes in principal components with SB204741 treatment that were nearly diametrically opposed in control and BMPR2 mutant mice (**Fig 5A**). Each principal component corresponds to a list of genes that are roughly co-regulated, with the first principal component (PC1) being the cluster of genes that explains the largest part of the variance across samples, PC2 being the gene group explaining the next most variance, etc [38]. The analysis was performed without identifying gene groups a priori; the grouping of the samples is thus a natural result of gene expression differences, rather than the result of selection. These data thus suggest opposite effects of drug in wild-type and BMPR2 mutant mice.

This differential effect can also be seen in a heatmap of the 100 genes most affected by SB204741, in which the direction of gene expression change is different in control and BMPR2 mutant mice (**Fig 5B, S1 Table**). When these 100 genes most affected by SB024741 are separated into statistically overrepresented gene ontology groups, the most statistically significant group is muscle contractility genes (**Fig 5C**), although there are additional metabolic, muscle structure, and cytoskeletal component groups that are also statistically overrepresented. Categories of genes are similar to those seen in the lungs of 5HTT-/- mice reported previously, although 5HTT-/- lungs also had changes in inflammatory and cell differentiation pathways not seen in inhibition of one receptor alone [39]. Ion channel genes were noticeably absent from the list of genes differentially regulated in these samples; this may be because they are lost in using whole lung, or because changes were functional rather than expression, or because the mechanism here is related to structure, rather than control, of the cytoskeleton.

**Fig 6:**
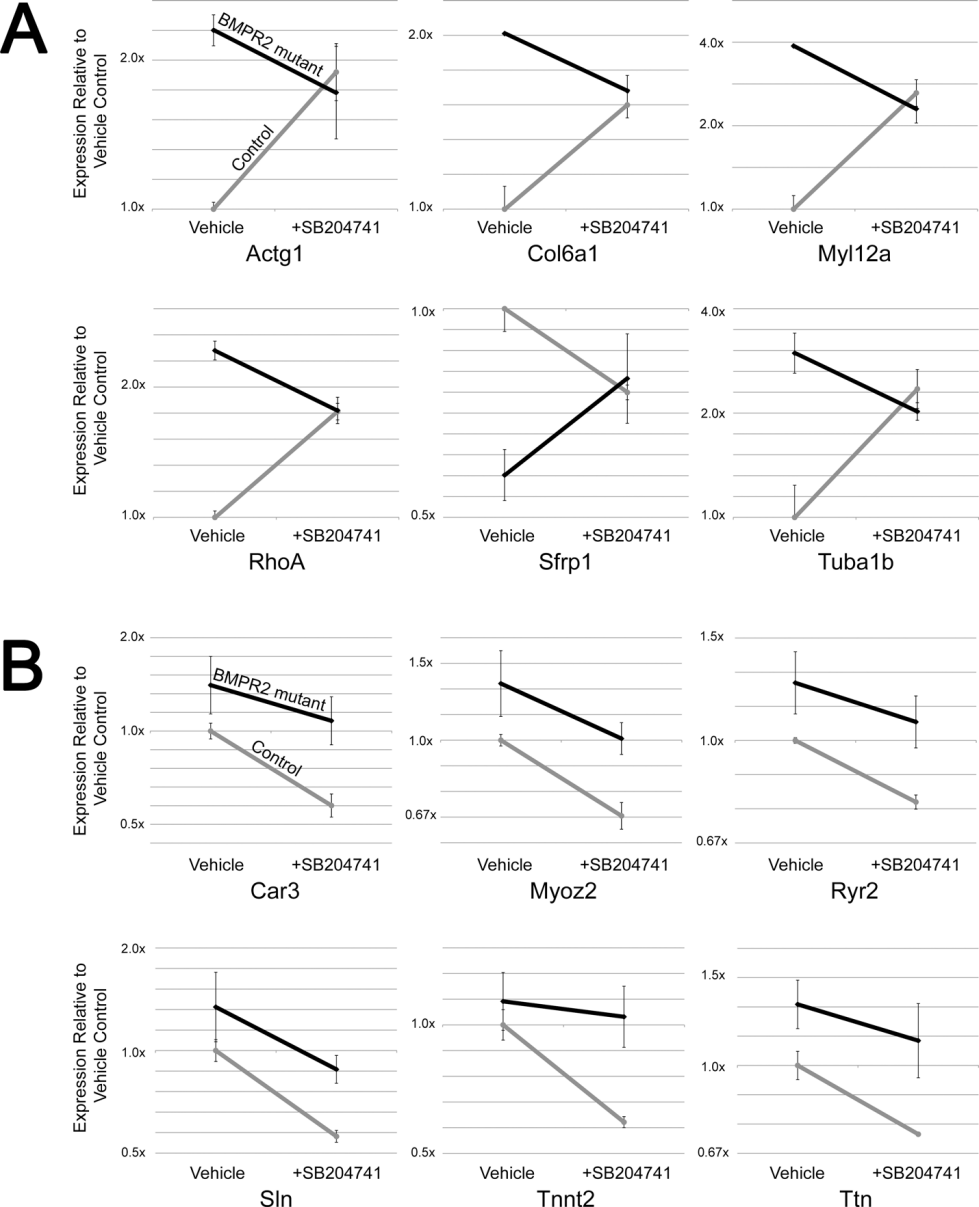
**(A)** SB204741 causes convergence of expression of most specific genes in the cytoskeletal component ontology group from Fig 5B between control and BMPR2 mutant lungs. **(B)** SB204741 results in reduced expression of most genes in the muscle contractility gene ontology group, which was the most statistically significant group in Fig 4B. This brings expression levels of BMPR2 mutant mice to control levels. Error bars are standard deviation. Grey lines are from control mice; black lines are from BMPR2 mutant mice in both A and B.

Some of the genes in these categories that are regulated in the opposite direction between BMPR2 mutant and control mice include contractility genes (RhoA, Gamma Actin, and Myosin Light Chain 12a) and microtubule trafficking genes (Tubulin α1b, Wnt inhibitor Sfrp1, and Collagen 6a1) (**Fig 6A**). However, there are additional muscle contractility and structure genes that are suppressed in both BMPR2 mutant and control cells, including a ryanodine receptor, titin, troponin t2, myozenin 2, carbonic anhydrase 3, and sarcolipin (**Fig 6B**). In summary, gene expression arrays on mouse lung indicate discrepant effects of SB204741 between control and BMPR2 mutant lungs, but with effects concentrating on muscle structure, contractility, and energetics. Note that levels of HTR2B was not different due to BMRP2 mutation or HTR2B antagonism.

### HTR2B Modulates Contraction in BMPR2 Mutant Smooth Muscle

**Fig 7:**
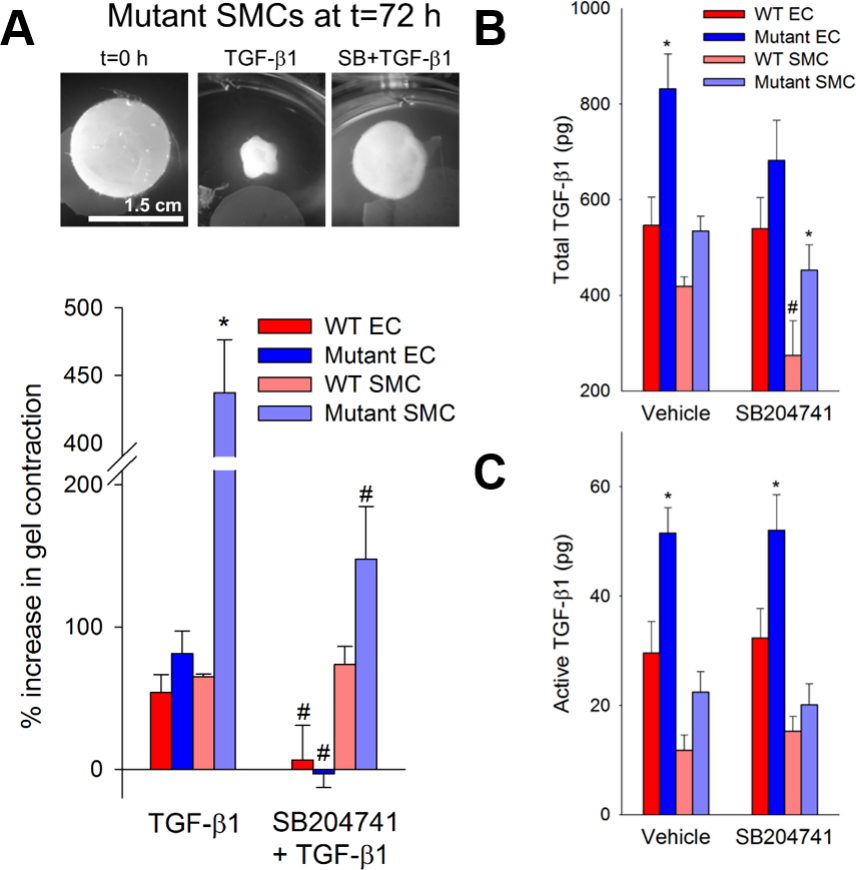
**(A)** SB204741 inhibits the elevated contractility of mutant microvascular cells in response to TGF-β1. Mutant microvascular smooth muscle cells exhibit a nearly five-fold increase in TGF-β1 induced contractility compared to their WT counterparts after 72 hours of treatment. TGF-β1 induced contractility in mutant microvascular smooth muscle cells is prevented when cells are treated concurrently with SB204741. Mutant cells synthesize **(B)** and activate **(C)** higher amounts of TGF-β1 than WT, and neither TGF-β1 synthesis nor activation is changed in mutant cells by SB204741. Values are expressed as mean ± standard error. n = 3–12 per group, *p<0.05 compared to WT, #p<0.05 compared to no treatment. Significance determined by a two-way ANOVA followed by a Holm-Sidak post hoc test.

To determine whether these observed gene expression changes could produce a functional outcome *in vitro*, both pulmonary microvascular endothelial cells and smooth muscle cells cultured from control and BMPR2 mutant mice were used in a gel contraction assay. Control and BMPR2 mutant endothelial cells had comparable levels of contraction in response to exogenously added TGF-β1; in both cases this was suppressed by incubation with SB204741 (**Fig 7A**). However, BMPR2 mutant smooth cells had approximately five times the level of contraction in response to TGF-β1 as did control cells, and while control smooth muscle cell contractility was not affected by SB204741, contractility in BMPR2 mutant cells was nearly normalized (**Fig 7A and S2 Fig**). Total and active TGF-β1 was also increased in both endothelial and smooth muscle cells from BMPR2 mutants but SB204741 did not appreciably alter these increases (**Fig 7B and 7C**), nor did the antagonist have any appreciable effect on smooth muscle cell proliferation (**S3 Fig**), suggesting rather direct effects on cytoskeletal remodeling.

## Discussion

**Fig 8:**
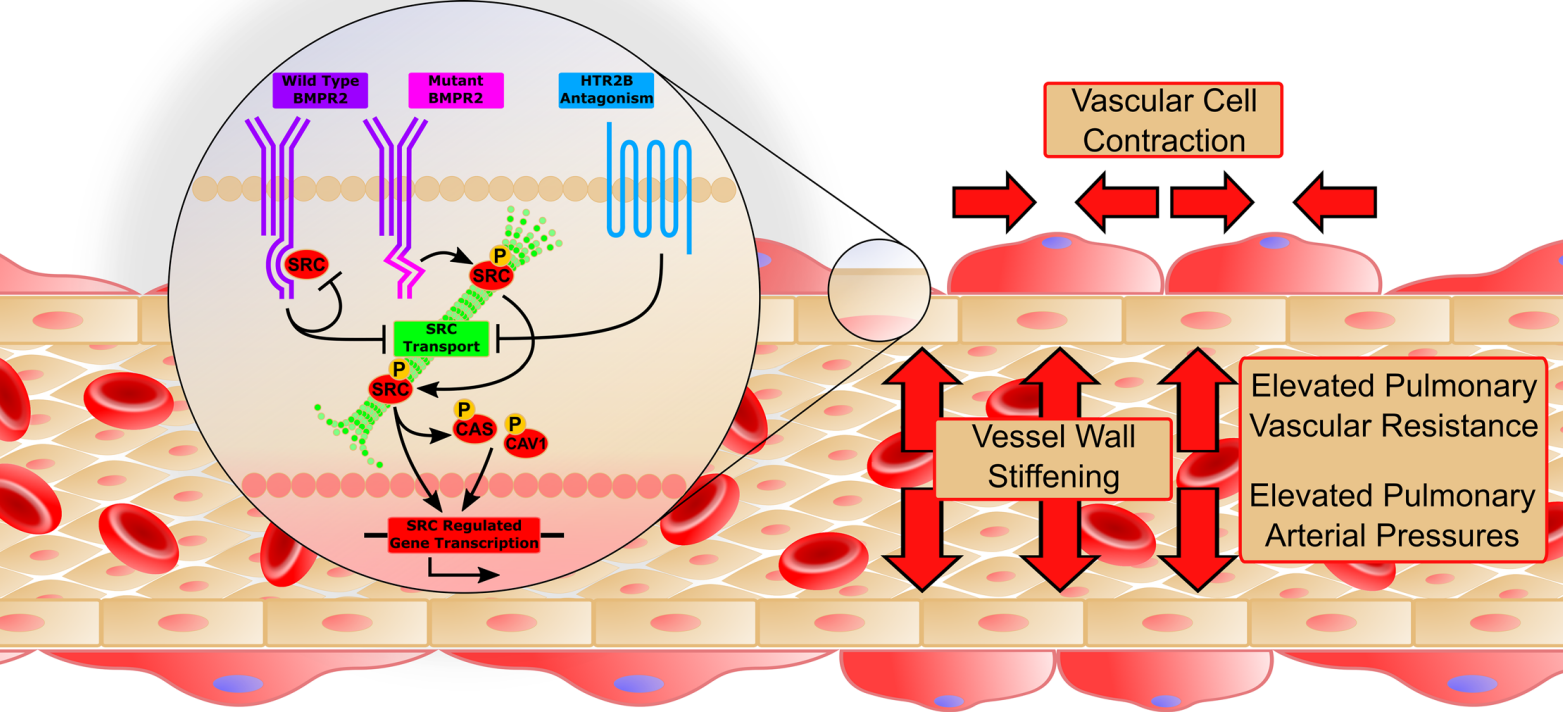
A proposed molecular mechanism for HTR2B antagonism to prevent heritable PAH. Mutations in the tail domain of BMPR2 result in increased SRC transport and signaling. Antagonism of HTR2B inhibits the translocation of SRC and decreases SRC signaling, causing a decrease in expression of SRC regulated genes. Functionally, this results in increased small vessel compliance, reduced inflammatory infiltrate, and decreased vascular smooth muscle contractility which together contribute to a restoration in mean pulmonary arterial pressures.

These results suggest that HTR2B antagonism can prevent the onset of heritable PAH by preventing the translocation and downstream activity of phosphorylated SRC due to BMPR2 mutation (**Fig 8**). Wild-type BMPR2 normally binds but does not phosphorylate SRC, with binding occurring in a long cytoplasmic tail that is unique to BMPR2 among TGFβ-superfamily receptors [14]. Mutations in the tail domain of BMPR2 results in an increase in both phosphorylation and downstream activity of SRC (**Fig 3A and 3B**) [15,37]. Here, we show that HTR2B antagonism prevents the BMPR2 mutation-mediated increase in SRC signaling (**Fig 3A and 3B**) through inhibition of SRC transport (**Fig 4A and 4B**). Further, inhibition of SRC translocation leads to modulation of cytoskeletal genes and functions through both direct (CAS and CAV1 mediated) [40] and transcriptionally regulated targets (**Figs 5 and 6**) [41]. Functionally, BMPR2 mutation leads to vascular stiffening *in vivo* (**Fig 2**), increased vascular cell contraction (**Fig 7**), increased inflammatory infiltration (**Fig 1C and 1D**) and elevated pulmonary vascular resistance (**Fig 1A**). This work thus demonstrates all of the elements present in **Fig 8**and establishes SRC activation as the primary target for preventing heritable PAH, and a strong candidate as the common signaling mechanism between drug-induced and heritable PAH.

One of the most interesting features of this data set is the finding that the effect of HTR2B inhibition is for many metrics completely opposite in WT and BMPR2 mutant mice. This includes vascular leak (**Fig 1C and 1D**), vessel stiffness (**Fig 2**), SRC motion (**Fig 4**), and patterns of gene expression (**Figs 5 and 6**). These strikingly discordant activities strongly suggest that the downstream signaling that arises from BMPR2 mutation and HTR2B agonism/antagonism is very direct (**Fig 8**). The most straightforward explanation of this is that SRC transport is dependent on its phosphorylation state or perhaps directly related to its BMPR2 binding.

BMPR2 mutation appears to alter TGF-β1 expression and activation in both endothelial and smooth muscle cells (**Fig 7B and 7C**), but HTR2B antagonism does not suppress this expression or activation appreciably. Thus, the mechanism of preventing cell contraction (**Fig 7A**) involves an intracellular target of the HTR2B receptor. Previously, we found that TGF-β1 ligand binding led to SRC phosphorylation directly from TGF-β1 type I receptor activation in heart valve cells [18]. Further, HTR2B antagonism prevented the downstream targeting of both CAS and p38 by TGF-β1-mediated SRC phosphorylation. In the current study, we see a similar response–HTR2B antagonism physically restricts SRC translocation and downstream activation of CAS and CAV1 (**Fig 3**) and this prevents BMPR2 mutation-induced vascular stiffening (**Fig 2**) and smooth muscle cell contraction (**Fig 7A**).

The ability of a HTR2B antagonist to prevent PAH by restricting downstream SRC activity (but not phosphorylation) calls into question the inability of receptor tyrosine kinase inhibitors, such as imatinib or nilotinib [42], to effectively treat PAH clinically. Presumably, these other inhibitors are non-specific, targeting multiple tyrosine kinases, and with their systemic delivery result in multiple alterations to signaling pathways that are important in maintaining cellular homeostasis in organs besides the lungs. Conversely, HTR2B offers a unique target for the treatment of PAH since it is largely restricted to the heart, lungs, liver, and gut with minimal expression in the brain and no known neurological function.

Although both serotonergic anorexigens and BMPR2 mutation are associated with PAH, it is important to note that the relative risk associated with BMPR2 mutations is much higher; roughly 100x for aminorex and 100,000x for BMPR2 mutation. One explanation for this dramatic difference in risk is that BMPR2 binds and signals through multiple mechanisms unrelated to SRC, including through LIMK, SMAD transcription factors, TCTEX1, and potentially other targets through binding to type 1 receptors [43]. These mechanisms each confer additional risk of PAH. For instance, loss of SMAD signaling results in smooth muscle cell transition to a synthetic state, with significant attendant vascular dysfunction [44].

It is instructive to compare our results with HTR2B antagonists in BMPR2 mutant mice with a recent study in which serotonin transporter (SERT) knockout was not protective against sugen/hypoxia induced PAH in rats [45]. Sugen/hypoxia can be thought of primarily as a model of severe endothelial damage with attendant remodeling, whereas although Bmpr2 mutants can develop significant endothelial lesions, these are rare and late in both mice [46] and humans [47]. This difference suggests that serotonin inhibition is not important in regulation of proliferation and remodeling, but rather plays an important role in initiating events and perhaps continuing underlying molecular pathologies.

While this study is the first to demonstrate a potential drug strategy for preventing heritable PAH in an animal model with the human-derived genetic mutation, it leaves several questions unanswered. In which cell type are these signaling defects most important? Vascular endothelium and smooth muscle, and a variety of circulating cell types are all potentially important targets [48]; the answer may be a combination of these. What are the intermediate systems through which HTR2B regulates SRC translocation? Moreover, because this was purely a prevention study, it is not clear that HTR2B antagonism would be capable of reversing established PAH. Further, because of the paradoxical effects of HTR2B antagonism in WT mice, it may not be a suitable point of intervention to correct the SRC defects in idiopathic PAH patients, although it may be beneficial in heritable patients. The present study, combined with existing literature showing that most of these defects are present in human PAH patients, suggests that this will be a viable therapeutic avenue, but multiple questions remain as to the best method and timing of intervention.

## Supporting Information

S1 Fig Weight change, cardiac output, gross lung architecture, and CD45 immunofluorescence. (DOCX)

S2 Fig Gel contraction time course data. (PDF)

S3 Fig BrdU positive cells. (PDF)

S1 Table Listing of 100 genes most affected by SB204741 treatment. (DOCX)

## Abbreviations

5HTT: serotonin transporter
αSMA: smooth muscle α-actin
AFM: atomic force microscope
CAS: p130Cas
CAV1: caveolin-1
HTR1B: serotonin 1B receptor
HTR2B: serotonin 2B receptor
BMP: bone morphogenetic protein
BMRP2: bone morphogenetic protein type II receptor
PAH: pulmonary arterial hypertension
RVSP: right ventricular systolic pressure
TMLC: transformed mink lung cell
TGF-β1: transforming growth factor β1
