# Supplementary figures and images for: Serotonin 2B Receptor Antagonism Prevents Heritable Pulmonary Arterial Hypertension
Source: PLoS One. 2016 Feb 10;11(2):e0148657. doi: 10.1371/journal.pone.0148657 (PMC4749293; doi:10.1371/journal.pone.0148657)

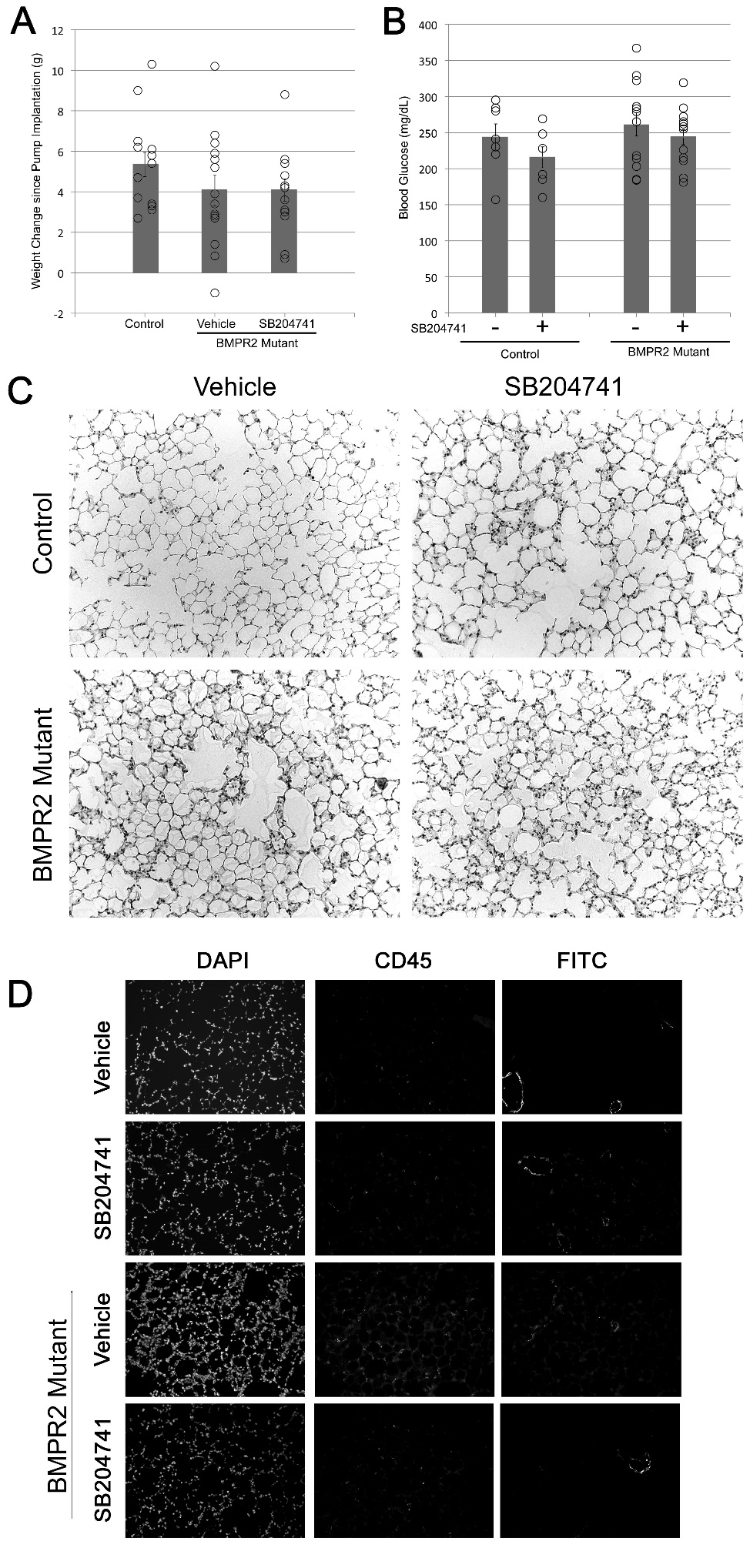


**Supplemental Figure 1: Weight change, cardiac output, gross lung architecture, CD45**

Supplement: S1 Fig — (DOCX) [file pone.0148657.s001.docx]

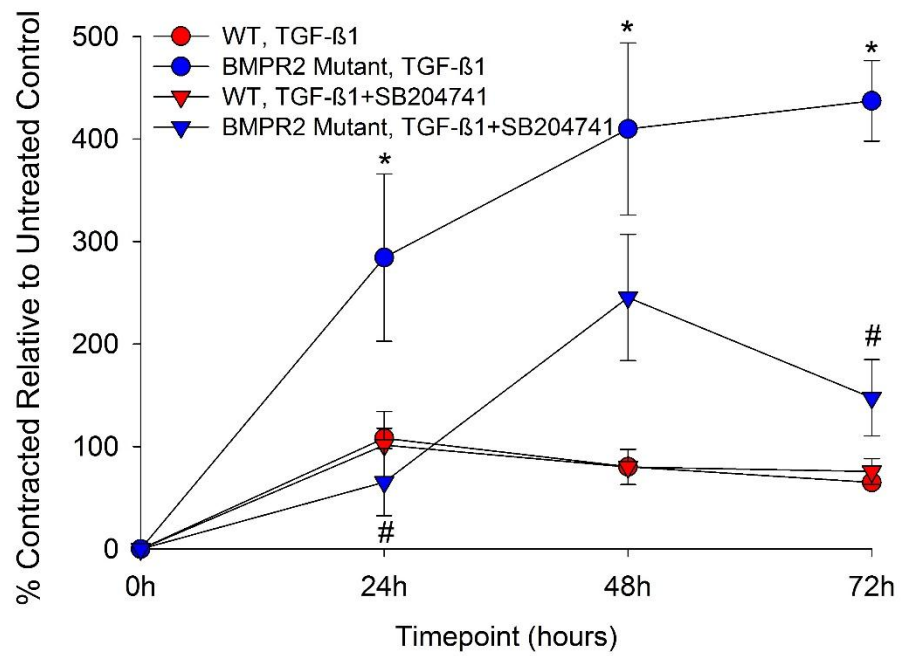

Supplemental Figure 2: Gel contraction time course data

Supplement: S2 Fig — (PDF) [file pone.0148657.s002.pdf]

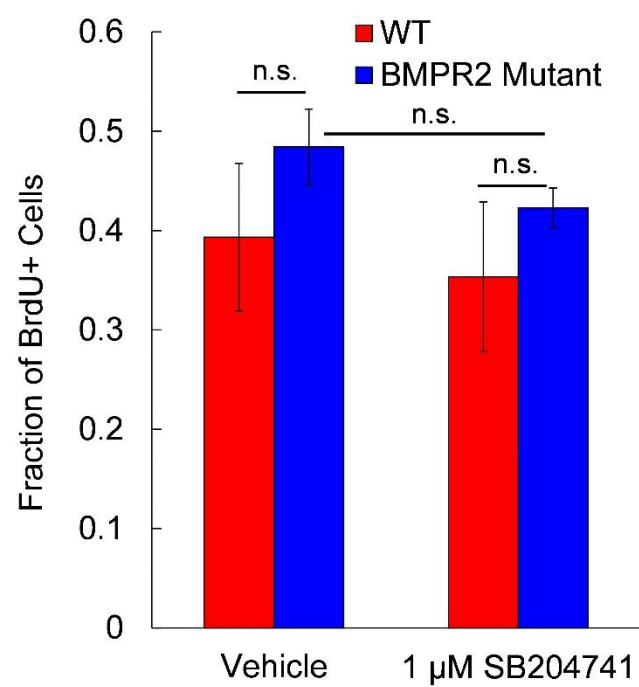

**Supplemental Figure 3: BrdU positive cells**

Supplement: S3 Fig — (PDF) [file pone.0148657.s003.pdf]
