# Supplementary material for: Serotonin 2B Receptor Antagonism Prevents Heritable Pulmonary Arterial Hypertension
Source: PLoS One. 2016 Feb 10;11(2):e0148657. doi: 10.1371/journal.pone.0148657 (PMC4749293; doi:10.1371/journal.pone.0148657)
Supplement: S1 Table — (DOCX) [file pone.0148657.s004.docx]

**Table 1: Listing of genes represented in heat map (Figure 5).**
